# Cancer-Associated Fibroblasts-Derived Exosomes as Mediators of Immunotherapy Resistance in Head and Neck Squamous Cell Carcinoma

**DOI:** 10.3390/cells14241978

**Published:** 2025-12-12

**Authors:** Julia Federspiel, Jozsef Dudas, Benedikt Gabriel Hofauer, Barbara Wollenberg, Teresa Bernadette Steinbichler

**Affiliations:** 1 Department of Otorhinolaryngology and Head and Neck Surgery, Medical University of Innsbruck, A-6020 Innsbruck, Austria; julia.ingruber@i-med.ac.at (J.F.);; 2 Department of Otorhinolaryngology, TUM Klinikum Rechts der Isar, Technical University of Munich, 81675 München, Germany

**Keywords:** PD-L1 inhibitors, exosomes, cancer-associated fibroblasts, EMT, tumor microenvironment

## Abstract

The tumor microenvironment (TME) orchestrates tumor growth, immune evasion, and therapeutic response in head and neck squamous cell carcinoma (HNSCC). Current immune checkpoint inhibitors (ICIs) target the programmed death receptor-1/programmed death-ligand 1 (PD-1/PD-L1) axis and improve survival in recurrent, metastatic, and locally advanced HNSCC. Tumor cells produced exosomes directly suppress cytotoxic T-lymphocytes activity by modulating immune checkpoint pathways and disrupting T-cell receptor signaling. Cancer-associated fibroblast-derived exosomes (CAF-Exos) function indirectly by conditioning immune escape and tumor growth. Together, these exosomal populations cooperate to create an immunosuppressive niche that hinders the efficacy of immunotherapies. CAF-Exos induce TME changes that exclude CD8^+^ T-cells, promote regulatory T-cells (Tregs), and upregulate PD-L1 expression in tumor cells. The bidirectional transfer of microRNAs (miRNAs) between tumor cells and CAFs enhances epithelial–mesenchymal transition (EMT), suppresses cytotoxic lymphocytes, and undermines ICI efficacy. This review article summarizes recent publications about plasma-derived exosomes from HNSCC patients. These exosomes carry tumor and immune checkpoint markers, reflect tumor burden and treatment response, and strongly modulate immune cells by suppressing T- and B-cell activity and promoting immunosuppressive macrophages. We encourage functional and biomechanistic future studies in the field of HNSCC that examine how CAF subtypes exosomes achieve an immunoresistant TME.

## 1. Introduction

HNSCC represents a highly heterogeneous group of tumors arising from the mucosal epithelium of the oral cavity, pharynx, and larynx. This heterogeneity encompasses diverse molecular characteristics, anatomical sites, and therapeutic responses. A central determinant of this diversity is the TME, a complex network composed of immune cells, stromal fibroblasts, endothelial cells, and extracellular matrix (ECM) components. Interaction between tumor cells and different fibroblast populations significantly determines outcome of therapies and immune reactions in the HNSCC tumor tissue.

Immune checkpoint inhibitor therapy has become an important option in head and neck cancer therapy, alongside surgery, radiation, and chemotherapy. Two immune checkpoint inhibitors (ICIs), nivolumab and pembrolizumab, have been approved based on clinical trials demonstrating improved survival outcomes of HNSCC patients [1,2,3].

Recently, pembrolizumab is gaining increased attention in patients with advanced stages of HNSCC as neoadjuvant and adjuvant treatment in addition to surgery and standard of care adjuvant treatment (radiotherapy ± cisplatin). In the KEYNOTE-689 trial, the addition of neoadjuvant and adjuvant pembrolizumab to standard of care significantly improved event-free survival among participants with locally advanced HNSCC [4,5]. The results of several trials are encouraging and suggest a specific function of immune checkpoint inhibitors in targeting micrometastatic or minimally residual head and neck cancer after curative surgical therapy that might be the source of postsurgical relapse [1]. Pembrolizumab and nivolumab target the PD-1/PD-L1 axis, a critical immune checkpoint pathway exploited by tumor cells to evade immune detection. PD-1, part of the CD28 receptor family, is expressed on activated T- and B-cells, Tregs, and natural killer (NK) cells. It binds to PD-L1, which is frequently overexpressed on tumor cells [3,6,7]. The PD-1/PD-L1 interaction enables tumor cells to evade immune cells mediated apoptosis by functionally “turning off” T-cells [8], allowing continued tumor growth. PD-L1 can be upregulated on tumor cells as an adaptive response to activated immune system such as the induction of interferon-γ in the TME [9,10]. The PD-L1/PD1 binding can induce apoptosis in active T-cells [11].

Despite treatment efficiency improvements by the neoadjuvant approaches, ICIs provide clinical benefit in only a subset of HNSCC patients (up to 44%) [12]. Limited efficacy is driven by primary and adaptive resistance within an immunosuppressive TME, including impaired interferon signaling, T-cell exclusion, and activation of alternative immune checkpoint pathways (LAG-3, TIM-3, TIGIT) [13,14,15]. Strategies that modulate the TME and restore interferon signaling are therefore of growing interest. Ribas et al. revealed that intratumoral oncolytic virotherapy induces a T-cell-inflamed TME, with increased CD8^+^ T-cell infiltration, PD-L1 expression, and type I interferon signaling, enhancing responsiveness to PD-1 blockade [14].

CAFs are essential factors in mediating primary resistance to immunotherapy. CAFs are associated with immune exclusion, CD8+ T-cell exhaustion, and increased regulatory CD4+ T cell infiltration, developing an immunosuppressive TME [16].

The adaptive resistance is a change in tumor cells, where the interaction with CAFs might play a role, but it is a protective response in tumor cells to the activated effector immune system [17]. Among stomal elements, CAFs are increasingly recognized as key drivers of immune resistance [18]. Recent evidence indicates that CAFs communicate with tumor and immune cells via exosomes [19,20], making these vesicles a novel focus of research.

CAF-Exos condition surrounding stromal and immune cells to support immune escape and tumor growth. Together with exosomes from other cellular sources, CAF-Exos cooperate to create an immunosuppressive niche that hinders the efficacy of immunotherapies [17]. Previous studies revealed that CAF-Exos delivered bioactive cargos, capable of inducing the gene expression of PD-L1 in tumor cells, thereby directly contributing to immune escape. The immunosuppressed niche in tumor tissue, which CAF-Exos significantly contribute to, might be related with primary resistance against immune checkpoint therapy [16,17,21]. Moreover, actual immune checkpoint inhibitor therapy itself can drive the development of secondary acquired resistance, a process in which CAFs and the exosomal vesicles produced by them might play important roles [22]. However, this mechanism remains largely unexplored.

Historically, the study of exosomes in cancer has progressed from the initial interpretation of extracellular vesicles as cellular “waste” in the 1980s, to their recognition as carriers of functional biomolecules in the 2000s, and ultimately to their current status as key mediators of TME communication and therapy resistance.

Exosome research includes their isolation from body fluids by differential ultracentrifugation, ultrafiltration, size exclusion chromatography, binding, and enrichment kits. The isolated exosomes are subjected to further analysis for RNA/miRNA, protein/mass spectrometry, and DNA [23]. While these techniques are well established and widely represented in the literature, they predominantly generate descriptive datasets and hypotheses. In contrast, experimental approaches from mono- and co-cultures or from organoids, which allow the production of exosomes and exosome-free fractions in a reproducible way, allowing biomechanistic findings, and rescue experiments are rare. A more challenging approach is the separation of exosomes released by different cellular sources, such as tumor cells and CAFs, which is essential for defining cell-specific effects [24].

## 2. Materials and Methods

This review was conducted in accordance with the PRISMA-ScR guidelines [25]. A comprehensive literature search was performed across the following electronic databases: PubMed, Web of Science, and Embase. The search covered all records from database inception up to June 2025. Search terms included combinations of “cancer-associated fibroblasts” or “CAFs”, “exosomes”, “head and neck squamous cell carcinoma” or “HNSCC”, “cancer”, “tumor microenvironment”, “immunotherapy”, “immune checkpoint inhibitors”, PD-L1, and “immune evasion”. Searches were limited to studies published in English and conducted in human subjects or involving in vitro/in vivo models of HNSCC. No restrictions were applied regarding publication year. Both original research and review articles were considered; however, preference was given to the most current and comprehensive studies. Earlier overlapping publications by the same research groups were excluded to avoid redundancy. Studies were included if they investigated CAFs and/or exosomes in the context of HNSCC; addressed molecular mechanisms, tumor progression, immunotherapy resistance, or clinical relevance; and used primary data from experiments, clinical samples, or validated models. Excluded were editorials, opinion papers, and studies lacking mechanistic or translational insight.

All references were managed using Zotero (Version 7.0, Corporation for Digital Scholarship (CDS) in Fairfax, VA, USA), and duplicates were removed prior to screening. Titles and abstracts were screened independently by the authors, and discrepancies were resolved by consensus. Full-texts articles were assessed for eligibility according to the predefined inclusion criteria. Potential sources of bias were minimized by applying predefined eligibility criteria, conducting independent dual screening, resolving discrepancies by consensus, and excluding overlapping publications from the same research groups.

In total, 176 records were identified and 26 duplicates removed, leaving 150 records for screening. After title and abstract screening, 133 full-text articles were assessed for eligibility, of which 71 were excluded. The first search (“CAF, exosomes, HNSCC, cancer”) yielded 52 papers, of which 15 were included (40 were excluded as out of scope). The second search (“tumor microenvironment, immunotherapy, immune evasion”) retrieved 66 references, with 27 retained. The third search (“immune checkpoint inhibitors, cancer-associated fibroblasts, HNSCC”) identified 58 papers, with 20 included. In total, 62 studies met the inclusion criteria and were included in the review. All included studies are summarized in Supplementary Table S1, providing the year of publication, first author, model type (in vitro, in vivo, or clinical), and key findings. To provide context and support for our findings, a total of 103 references are cited throughout this manuscript. No prior protocol was registered for this review. The overall process of study identification, screening, eligibility assessment, and inclusion is summarized in the Prisma-ScR flow diagram (Supplementary Figure S1). Comparative insights as “CAF-exosomes, difference, tumor-derived exosomes” were added.

## 3. Exosomes in CAF-Tumor Crosstalk and Immune Regulation

### 3.1. Exosomes Biogenesis and Composition

Exosomes are a class of extracellular membrane vesicles with a size ranging from 40 to 150 nm [26]. Exosomes enable intercellular communication without direct cell–cell contact. They can be secreted by epithelial, tumor, and stromal cells. Exosomes can be identified by their size, round or cup-shaped morphology, and density between 1.13 and 1.19 g/mL [27,28]. They are characterized by their unique protein and lipid composition and double lipid layer due to their origin in the fusion of late endocytic compartments with the plasma membrane [29]. The increased secretion of exosomes by tumor cells is induced by the overexpression of Rab3D [30]. The acidic microenvironment of tumors stimulates the release of exosomes and enhances their cell fusion capabilities [31]. Release of exosomes can also be induced by diverse signaling pathways. The WNT and MAPK signaling pathways, for example, are involved in exosomal transport and serve as therapeutic targets [32].

The uptake of exosomes is accomplished via endocytosis, receptor–ligand interaction, or by direct fusion, and is dependent on microenvironmental pH. Exosomes transport double-stranded DNA, proteins (metabolic mediators and signaling components), and especially RNA (mRNAs, microRNAs, long non-coding RNAs) from one cell to another, causing alterations in gene expression of the recipient cell [33]. In HNSCC, exosomes contribute to therapy resistance by disseminating anti-apoptotic signals, enhancing DNA repair mechanisms, and delivering ATP-binding cassette (ABC) transporters to otherwise drug-sensitive cells [34,35]. In addition to their therapy-resistance supporting effects, several reports from Whiteside T. and colleagues evidenced substantial functions of tumor cells-derived exosomes (TEXs) in the suppression of anti-tumor function of cytotoxic T-lymphocytes, especially in HNSCC [19,20].

**Figure 1:**
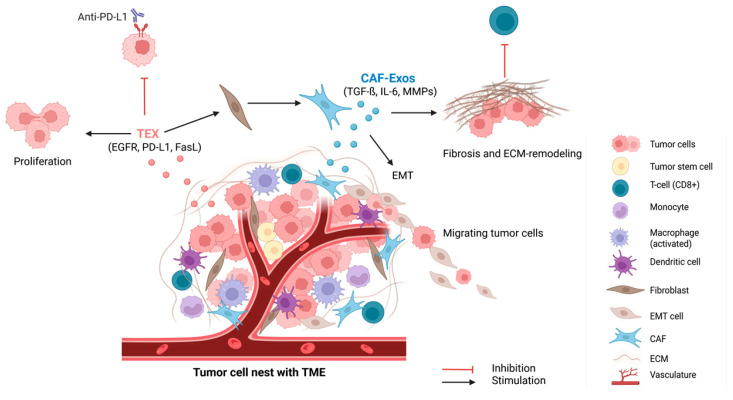
Cooperative immunosuppression mediated by tumor cells, CAFs, and their exosomes. TEXs and CAF-Exos remodel the TME to promote immune evasion and resistance to immune checkpoint blockade. TEXs directly suppress cytotoxic CD8^+^ T-cell function via PD-L1/PD-1 and FasL signaling and promote tumor cell proliferation through EGFR-mediated pathways. In addition, TEXs can activate NF into CAFs. Activated CAFs release exosomes containing proteins such as TGF-β, IL-6 and MMPs, driving EMT, fibrosis, and ECM remodeling. Altered ECM architecture prevents CD8^+^ T-cell infiltration into the middle of the tumor cell nest. Collectively, the TME and the containing TEXs and CAF-Exos sustain a protumorigenic and immunosuppressive microenvironment that hinders the efficacy of anti-PD-L1 immunotherapy. Created in BioRender. Dudas, J. (2025) https://BioRender.com/irtrlo8 (accessed on 29 October 2025).

TEXs directly suppress cytotoxic T-lymphocytes activity by modulating immune checkpoint pathways and disrupting T-cell receptor signaling [36]. In contrast, CAF-Exos often function more indirectly by conditioning other stromal and immune cells to support immune escape and tumor growth. Together, these exosomal populations cooperate to create an immunosuppressive niche that hinders the efficacy of immunotherapies. While both TEXs and CAF-Exos carry proteins, lipids, DNA, and RNA species, their cargo reflects their characteristics of their cell of origin. TEXs are rich in immunosuppressive and tumor-promoting proteins like PD-L1, FasL [37], and heat shock proteins such as HSP70 [38] and HSP90 [39], which help tumor cells to evade immune surveillance. TEXs often carry the human epidermal growth factor receptor (EGFR), supporting proliferation and resistance to the EGFR-targeted therapy (cetuximab) in HNSCC [40,41]. CAF-Exos are enriched in stromal-modulating proteins like transforming growth factor-beta (TGF-β), Interleukin-6 (IL-6), and Matrix metalloproteinases (MMPs), promoting fibrosis, ECM remodeling, and an immune-excluded tumor phenotype (Figure 1).

CAFs secrete exosomes that enhance the gene expression of PD-L1 in tumor cells, independent of immune activation. This prepares the tumor cells to be protected against immune attacks. The corresponding paper was about breast cancer. This paper did not detail which cargo in the CAFs’ exosomes was responsible for the increased expression of miR-92 in CAFs’ exosome-treated breast cancer cells. Nevertheless, miR-92 seems to play a central role in increasing PD-L1 expression via the following pathway: miR-92 targets LATS2, which interacts with YAP1. YAP1 then binds to the enhancer region of PD-L1, which subsequently upregulates PD-L1 [42]. We investigated the available literature to determine if this seemingly important pathway in CAF exosomes, which induces tumor PD-L1 expression, is also relevant and has been significantly studied in head and neck cancer. However, no similar articles were found, except for the aforementioned breast cancer paper. A previous review article investigated “The Hidden Link of Exosomes to Head and Neck Cancer” and cited breast cancer as an example in mechanistic discussions [43]. This review article encourages functional and biomechanistic original studies in the field of head and neck cancer, which examine how CAF exosomes make HNSCC tumor cells immunoresistant, a perspective that our research group will also adopt. In addition, the enhancement of alternative immune checkpoints, especially after PD-1 antibody therapy, is an important resistance mechanism after treatment with pembrolizumab. However, the biomechanical aspects of CAF–tumor cell exosome exchange in relation to alternative immune checkpoints activation have not yet been investigated. Here, the knowledge of gene expression and descriptive studies must be turned into biomechanistic experimental works.

### 3.2. Experimental Approaches for Isolating CAF-Exos and TEX

There are various methods for isolating exosomes from tumor tissue, body fluids like blood or saliva, and cell culture media. Differential ultracentrifugation is simple and widely used but often yields preparations contaminated with proteins and nucleic acids, whereas ultrafiltration is faster and suitable for large volumes but can damage the exosomes by high pressure. Density gradient centrifugation and size exclusion chromatography provide higher purity and structural integrity, though they require specialized equipment and may have lower yields. Polymer precipitation and immunomagnetic bead separation offer efficient or highly specific isolation, respectively, but are limited by the need for known surface markers and therefore carry a risk of a selection bias [23].

Investigating tumor cells and CAF-Exos can be achieved by culturing these cells separately, isolating exosomes, and examining them individually or in combination. Another option is to culture tumor cells and fibroblasts together and isolate the exosomes from the mixed cultures. A more challenging, yet more realistic, approach is to separate tumor cells and CAF-Exos from blood, saliva, patient-derived organotypic tissue cultures, or organoids. In this case, the separation of the exosomes can mainly be based on the use of immunomagnetic beads that are specific to CAF or tumor cell markers. This method could enable the removal of either tumor cells or CAF-Exos [24].

### 3.3. CAFs Heterogeneity and Immunosuppressive Mechanisms

CAFs are a heterogeneous population consisting of multiple subtypes with distinct phenotypic and functional profiles [44,45]. This diversity enables specialized roles in tumor progression, immune modulation [46], and response to therapy [47,48,49]. The CAF compartment in HNSCC consists of four major subclusters, as observed in breast, urothelial, or pancreatic cancer. The first is a myofibroblastic (my)CAF-like cluster, which aligns with classical myCAF signatures and represents the main source of transcriptional differences between tumor-derived and normal fibroblasts. This population is the dominant CAF subset located around tumor nests, as confirmed by spatial transcriptomics, and plays a central role in restricting CD8^+^ T-cell infiltration into the tumor core. The second subcluster is an inflammatory (i) CAF-like population, characterized by inflammatory and cytokine-rich features consistent with previously described iCAF signatures. The third is a smaller hybrid or transitional cluster that co-expresses markers of both myCAFs and iCAFs, suggesting a dynamic state between these two major CAF phenotypes [44]. Finally, a fourth cluster of antigen-presenting CAFs (apCAF), arising predominantly from myeloid cells through MIF-driven JAK/STAT3 signaling, induces an unfavorable CD4+/CD8+ T-cell balance, fosters an immunosuppressive environment, and accelerates tumor progression [50]. Common CAF markers include alpha-smooth muscle actin (α-SMA), Podoplanin (PDPN) fibroblast activation protein (FAP), and platelet-derived growth factor receptors (PDGF) [44,51]. MyCAFs are characterized by high α-SMA and PDPN expression and contractile activity; iCAFs secrete cytokines such as IL-6 and C-X-C motif chemokine ligand 12 (CXCL12) to modulate immune responses; and an apCAF population expresses MHC class II molecules but lacks classical co-stimulatory markers [47].

Although all CAF subsets release exosomes, single-cell and spatial transcriptomic analyses consistently show that iCAFs exhibit the strongest secretory and vesicle-export gene programs [17]. Through their paracrine activity, iCAFs secrete cytokines, chemokines, and exosomes that modulate immune cells and promote tumor cell proliferation in solid tumors, including HNSCC. MyCAFs, while less secretory than iCAFs, still produce relevant levels of exosomes that support tumor cell migration, ECM remodeling, and metastatic dissemination. In contrast, apCAFs appear to have the lowest exosome output and primarily influence T-cell activity, contributing to immune tolerance [48].

One critical function of CAFs is their capacity to suppress anti-tumor immune responses. The secretion of immunomodulatory cytokines, chemokines, and growth factors, such as TGF-β, IL-6, and CXCL12, inhibits cytotoxic T-cell activity and promote the recruitment and expansion of Tregs, thereby fostering an immunosuppressive microenvironment [52,53]. Tregs contribute to immune evasion by inhibiting the activity of cytotoxic CD8^+^ T-cells and other immune effector populations. They suppress immunity by releasing cytokines like IL-10 and TGF-β [54], expressing checkpoint molecules such as CTLA-4, and modulating metabolism through IL-2 consumption and adenosine production [55]. In HNSCC, elevated infiltration of Tregs correlates with impaired anti-tumor immunity and poor clinical outcomes [56].

In addition, Tregs and NK cells can be directly suppressed by CAFs through the secretion of PD-1 and CXCL5 [57]. CXCL5 recruits myeloid-derived suppressor cells (MDSCs) through its receptor CXCR2, thereby inhibiting NK and T-cell infiltration and promoting a T-cell-excluded tumor phenotype [58,59]. Elevated CXCL5 expression has been correlated with reduced CD8^+^ T-cell presence and poor responses to PD-1 blockade in several cancer types, including HNSCC [60].

Additionally, CAFs can transfer PD-L1 via exosomes or stimulate its upregulation in tumor and immune cells. Exosomal PD-L1 can directly bind to PD-1 on T-cells, causing T-cell exhaustion and inhibition of cytotoxic responses. This mechanism contributes to resistance against PD-1/PD-L1 inhibitors [61,62].

Mito et al. provided direct evidence for the role of TEXs from HNSCC cell lines in mediating immunosuppression and upregulation of classic CAF markers [63]. Their data demonstrated the suppression of CD4^+^ and CD8^+^ T-cell proliferation and the induction of M2-like pro-tumoral macrophage phenotype via exosome-educated fibroblasts. RNA-sequencing of exosome-educated fibroblasts revealed the activation of the IL-6 and IL-17 signaling pathways [63]. The reported activation of IL-17 signaling in CAFs educated by tumor exosomes is less often discussed in the exosome-CAF literature. It suggests a specific Th17/IL-17-driven inflammatory axis in HNSCC, amplifying neutrophils and MDSC, which reinforce immune evasion.

It is well documented that CAFs promote EMT [61,64], leading to enhanced PD-L1 expression in HNSCC in vivo and vitro models [65]. EMT is a highly dynamic process where epithelial tumor cells acquire mesenchymal features, enhancing their migratory and invasion potential. Molecular characteristics, relevant EMT markers in HNSCC, and their clinical relevance have been investigated by our group and published elsewhere [66,67,68].

Adding to their immunosuppressive repertoire, CAFs have been shown to express SerpinB9, a serine protease inhibitor that blocks granzyme B-mediated cytotoxicity. This allows them to evade immune-mediated cell death while simultaneously protecting neighboring tumor cells from cytotoxic lymphocyte-induced apoptosis. SerpinB9 is an inhibitor located between the activated granzyme B and the caspase cascade. The inhibitory loop of serpins acts as a pseudosubstrate for granzyme B and forms an irreversible complex with it following cleavage [69]. The presence of SerpinB9-expressing CAFs thus enhances tumor cell survival and facilitates immune evasion [70], contributing to a more aggressive and therapy-resistant tumor phenotype.

**Figure 2:**
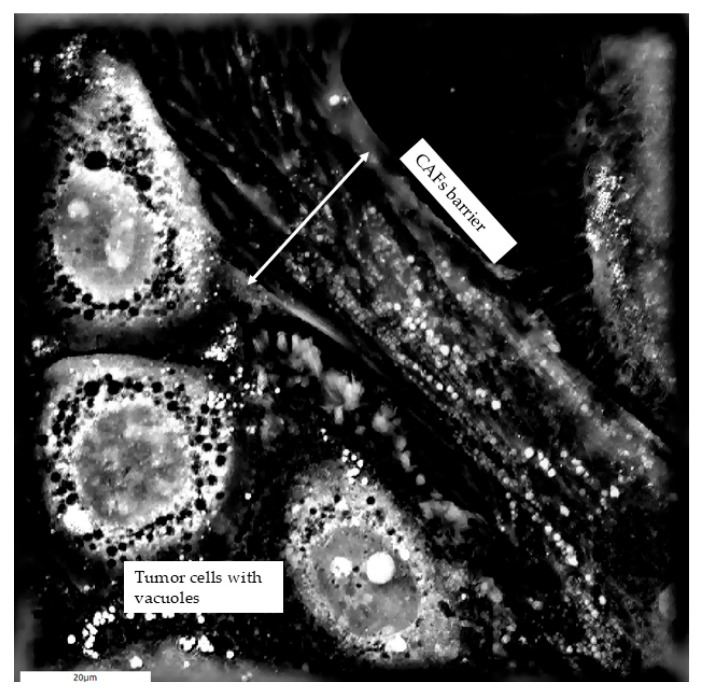
Holotomographic image showing SCC-25 cells in vitro containing multiple small, dark intracellular vacuole-like structures. Surrounding CAFs form a dense physical barrier around the tumor cell clusters, highlighting their potential role in spatial confinement and protection of tumor cells within the TME. Adapted from [62] under the Creative Commons Attribution (CC BY) license. Scale bar: 20 µm.

Furthermore, CAFs act as physical barriers to immune filtration and therapeutic agents. CAFs form a contractile capsule around the tumor cell nest through the excessive deposition of ECM components, leading to increased interstitial pressure of tumor cells and restricting immune cell access [57,62] (Figure 2). This barrier function may, under certain conditions, transiently limit tumor cell proliferation by limiting nutrient diffusion and mechanical expansion [62,71]. Simultaneously, CAFs actively promote tumorigenesis by secreting growth factors such as EGF, HGF, and TGF- β that stimulate tumor cell proliferation, EMT, and survival [72].

### 3.4. CAF-Exos and ECM Components

CAF-Exos might deliver ECM components or carry regulators enhancing ECM stiffness increases. The lower availability of tumor area to killer immune cells accompanied by lack of immune infiltrate is characterized by insufficient immune response and cannot be repaired by immunotherapy approaches [73]. CAFs achieve senescent phenotype upon the interaction with tumor cells, and these CAFs are highly engaged with the tumor-capsulating matrix deposits [74,75]. The therapeutic targeting of CAFs is based on their special metabolic characteristics, which will be probably required to add to immunotherapeutic approaches. This idea is presented in a current paper of Wu et al. The suppression of GLUT-1-based lactate-rich glycolytic metabolism in CAFs resulted finally in more effective anti-tumor immune functions [73].

Although CAFs are widely recognized for promoting tumor progression in HNSCC, CAF secretome can also have anti-tumor effects, depending on stage of tumorigenesis and microenvironmental cues. CAF-derived TGF-β may suppress tumor growth by inducing epithelial cell cycle arrest and apoptosis, thereby maintaining tissue homeostasis, which can restrain tumor initiation in the early tumor stage. However, in advanced stages, the initially tumor-suppressive role of CAF-derived TGF-β is subverted, as tumor cells exploit the growth factors secreted by CAFs to enhance their own proliferation and migratory potential [76,77].

### 3.5. CAF-Mediated Immune Exclusion Correlates with ICI Resistance, Clinical Relation

A clinically important recent approach is recognizing the significance of the CAF marker FAP in relation to prognosis and predicting immunotherapy response, as well as its usefulness as a PET tracer and in developing a therapeutic approach of radionuclide conjugates. These studies establish the foundation for understanding the clinical significance and patient care implications of CAF immune exclusion functions. Single-cell analysis of breast cancer revealed a positive feedback loop between cancer-associated fibroblasts (FAP^+^ CAFs) and Tregs that contributes to immunotherapy resistance [78].

Fabre et al. further proved the significant role of FAP^+^ CAFs in immunotherapy resistance [79]. They produced an antibody-drug conjugate that targeted FAP. Using a mouse model with a humanized immune system that is resistant to PD-1 inhibition, they found that the conjugate increased tumor infiltration by CD8+ T-cells, induced complete regressions, and delayed tumor recurrence. This provided further preclinical evidence of the central role of targetable CAFs in ICI therapy resistance. A further preclinical in vivo study revealed that [^225^Ac] Ac-FAPI-46 enhances immune checkpoint blockade, and its efficacy correlates with tumoral FAP expression levels [80].

An interesting clinical study is the phase Ib trial evaluating the immunocytokine FAP-α-targeted IL2 variant (FAP-IL2v). FAP-IL2v was combined with cetuximab in patients with recurrent, unresectable, or metastatic HNSCC.

FAP-IL2v preferentially expanded intratumoral NK and CD8+ T-cells. This is clinical evidence of the important role of CAFs in immunosuppression and the lack of an effective immune response in HNSCC tumors. The trial is in the early phase, but four patients achieved a partial response, and the objective response rate was 7% [81].

### 3.6. Exosomal miRNAs Mediate Bidirectional Communication Between CAFs and Tumor Cells

Crosstalk between CAFs and exosomes forms a feed-forward loop that enhances tumor-supportive conditions within the TME. TEXs can induce the differentiation of normal fibroblasts into activated CAFs by transferring oncogenic proteins, miRNAs, and TGF-β–containing vesicles, leading to enhanced secretion of ECM proteins, cytokines, and growth factors that reinforce a pro-tumorigenic microenvironment. In parallel, CAF-Exos modulate tumor cell behavior, supporting tumor growth and survival pathways.

MiRNAs play pivotal roles in exosome-mediated tumor–stroma interactions in HNSCC (Table 1). Under hypoxic conditions, HNSCC cells upregulate hypoxia-inducible factor-1α (HIF-1α), which transcriptionally activates miR-5100 [82] and miR-21 [83]. These miRNAs are selectively packed into exosomes and transferred to surrounding fibroblasts. In recipient fibroblasts, miR-5100 directly targets and downregulates Quaking (QKI), an RNA-binding tumor suppressor in HNSCC. Loss of QKI leads to activation of the AKT and STAT3 signaling pathways, resulting in increased α-SMA expression, cytokine secretion, and ECM remodeling. Inhibiting AKT/STAT3 reverses α-SMA induction, showing a direct pathway to CAF differentiation [82].

Similarly, exosomal miR-21 activates SMAD-dependent signaling and Wnt/β-catenin pathways [84], driving EMT in tumor cells and the differentiation of bone marrow-derived mesenchymal stem cells into functional CAFs. This process is driven by miR-21 suppression of target genes such as PTEN and PDCD4, leading to increased α-SMA and fibronectin expression. In turn, activated CAFs secrete exosomes and soluble factors such as IL-6, CXCL12, and WNT-ligands, which induce JAK/STAT3 signaling in tumor cells and an enhanced PD-L1 expression, supporting immune evasion. Furthermore, exosomal miR-21–mediated activation of STAT3 promotes phosphorylation of p38 MAPK via the non-canonical TGF-β signaling pathway, particularly under conditions of cellular stress. In 2023, our group demonstrated that repeated TGF-β treatment activates p38 MAPK, stabilizes the transcription factor Slug, and induces partial EMT in HNSCC cells [67]. The miRNA-driven stromal activation not only promotes tumor dissemination but also impairs anti-tumor immunity and therapeutic response [85]. As discussed by Ye et al. (2023), circulating exosomal miRNAs, including miR-21, show potential for monitoring metastatic risk and treatment resistance [83].

CAF-Exos carry miRNAs, including miR-196a, miR-215, and proteins that promote EMT, stemness, and therapy resistance in tumor cells [86,87]. CAF-Exos enriched with miR-196a have been shown to confer cisplatin resistance by targeting tumor suppressors such as CDKN1B and ING5 in HNSCC cells. Inhibition of miR-196a in these exosomes restores chemosensitivity and correlates with improved clinical prognosis [87]. Hypoxia-induced miR-215 has been implicated in enhancing tumor cell survival by targeting key tumor suppressors such as RB1 and modulating p53-mediated DNA damage response mechanisms, thereby contributing to chemoresistance. Moreover, TEXs enriched with miR-192/215 suppress Caveolin-1 in normal fibroblasts. This triggers CAF-like differentiation via activation of the TGF-β/SMAD pathway, creating a pro-invasive microenvironment [88].

Exosomes secreted by CAFs differ in characteristics and biological functions from those from normal fibroblasts. Wang et al. demonstrated that CAFs secrete exosomes with significantly reduced levels of miR-3188 compared to those derived from normal fibroblasts. In vitro experiments and in vivo validation showed that reduced miR-3188 in CAF-Exos upregulates BCL2 in HNSCC cells, promoting increased proliferation and reduced apoptosis. Conversely, treatment with miR-3188-enriched exosomes inhibited tumor growth in xenograft models, highlighting the therapeutic potential of miR-3188 delivery in HNSCC [89].

### 3.7. Tumor Cells Independent Direct Effects of CAFs on Effective Immune Cells via Exosomes

Immune functions are controlled by CAFs via constitutive secretion of cytokines, chemokines, growth factors, and ECM proteins. In addition to the direct release into the surrounding ECM, influences through micro-vesicles play increased roles. A further form of the direct influence of CAFs on effective immune cells is that CAFs express key regulators of immune checkpoints [46].

CAFs interact with macrophages and dendritic cells (DCs) to establish an immunosuppressive microenvironment. This, in turn, indirectly impairs T-cell-mediated anticancer immunity, facilitating immune evasion. In addition, CAFs also exert direct suppressive influences on the effector functions of T-cells. This is mainly achieved by ECM remodeling and promoting the expression of immune checkpoints, cytokine secretion, and the release of extracellular vesicles. The main consequence is the impaired T-cell proliferation, increased T-cell apoptosis, prevented migration and infiltration, changes in differentiation and induction, and T-cells exhaustion [90].

Obradovic et al. highlight a novel mechanism of resistance to anti-PD-1 immunotherapy in HNSCC, whereby a specific CAF subpopulation, particularly HNCAF-1, fosters a local immunosuppressive microenvironment that hinders T-cell functionality. The HNCAF-1 subtype was predominantly found in non-responding tumors and exhibited immunosuppressive properties. Spatial transcriptomics showed its co-localization with exhausted CD8^+^ T-cells, marked by elevated PD-1 and TIM-3 expression. In co-culture assays, HNCAF-1 cells induced T-cell exhaustion, reduced cytotoxic molecule expression, and impaired memory T-cell development. In contrast, HNCAF-0 and HNCAF-3 subtypes promoted T-cell activation and were linked to better ICI response. These findings suggest that CAF subtypes influence immunotherapy outcomes and may serve as predictive biomarkers or therapeutic targets [91].

In different cancers, the main exosomes-based role of CAFs’ immunosuppressive effects relies on delivery of immune checkpoint molecules or orchestrates an expressional background for increase in immune checkpoint molecules, induction of exhaustion, and inhibition of CD8^+^ T-cell proliferation [92].

An interesting example is the regulatory mechanism of CAF-Exos, which, after intermediate steps, enhance the nuclear translocation of yes-associated protein 1 (YAP1) in exosome target cells, as in effector immune cells. In this way, YAP1 binds to the enhancer region of PD-L1 to promotes its transcriptional activity [42].

## 4. Impact of HNSCC Patient-Derived Exosomes on Immunotherapy

Plasma- and saliva-derived exosomes from HNSCC patients exhibited typical vesicular morphology (30–200 nm) and expressed canonical exosomal markers such as CD9 and CD63, suggesting that they are at least in part secreted by CAFs. Compared to healthy donors, HNSCC-derived exosomes contained higher levels of immune checkpoint molecules (TIM-3, PD-L1) and tumor-associated markers (CD44v3, CD39). The protein content of plasma exosomes correlated with tumor burden and changed dynamically during therapy, reflecting treatment response and disease progression [93,94,95].

All HNSCC patient-derived exosomes also expressed CD16 on their surface, with higher levels observed in patients with advanced UICC stages. In contrast, CD44v3^+^ exosomes—used as tumor markers—showed lower CD16 expression, suggesting that CD16 primarily originated from immune cells rather than tumor cells. The abundance and size distribution of CD16^+^ exosomes correlated with CD16^+^ non-classical monocytes and adhesion molecules such as CD29 (integrin β1) and CX3CR1 on specific monocyte subsets [96,97]. Plasma-derived exosomes from HNSCC patients exhibited strong immunomodulatory effects on T-cells. CD45(−) exosomes (tumor-enriched exosomes) induced apoptosis of activated CD8^+^ T-cells, suppressed their activation, promoted the differentiation of CD39^+^ Treg cells, and generated immunosuppressive adenosine, whereas CD45+ exosomes had weaker, stage-independent effects. Moreover, CD45(−) exosomes from patients with high UICC stages induced stronger CD8^+^ T-cell apoptosis compared to those from patients with early-stage disease. Co-culture of immune cells with exosomes inhibited B-cell proliferation, survival, checkpoint receptor expression (including CD39), and B-cell receptor signaling. Uptake of exosomes by monocyte-derived macrophages resulted in M2-like polarization, upregulation of PD-L1, and increased CXCL4 secretion, thereby promoting tumor cell migration while suppressing cytotoxic T-cell activity [98,99,100].

Plasma-derived exosomes collected from HNSCC patients before and after therapy also had the ability to mediate therapy-induced EMT modulation in vitro. Exosome-induced changes in cell migratory potential served as discriminators of treatment outcome. Furthermore, plasma derived exosomes reflected tumor burden and treatment response: treatment-naive exosomes induced EMT in vitro, whereas exosomes from patients with a surgical treatment caused epithelial characteristics and exosomes from conservative treated patients caused mesenchymal traits and increased Twist/Slug expression [101]. HNSCC exosomes inhibited endothelial cell proliferation, migration, and tube formation while promoting apoptosis; these effects decreased with increasing tumor stage. Plasma exosomal Arginase-1 (Arg-1) is associated with lymph node metastasis and poor prognosis, whereas intratumoral Arg-1 correlated with better outcomes. Overall, HNSCC-derived exosomes shaped the tumor microenvironment, modulated systemic immunity, and served as promising biomarkers for diagnosis, prognosis, and therapy monitoring [102].

Exosome isolation studies have limitations because the most common methods of isolation, such as ultracentrifugation, density-gradient centrifugation, and precipitation, do not meet the requirements for a fast, high-throughput method of isolating pure and abundant exosomes. Nevertheless, to ensure comparable exosome preparation quality and purity across studies, the Minimal Information for Studies of Extracellular Vesicles (MISEV) guidelines provide criteria for defining EVs and offer recommendations for experimental setups and data interpretation [103]. Another limitation of the aforementioned published works is their relatively small sample size of 21–32 patients. While the sample size may increase random effects, adherence to the aforementioned guidelines increases reproducibility.

## 5. Conclusions

HNSCC is driven by an immunosuppressive TME that limits the efficacy of ICIs. Beyond secreting soluble factors, CAFs actively remodel the tumor immune microenvironment through exosome-mediated delivery of PD-L1, TGF-β, IL-6, CXCL12, and matrix metalloproteinases, directly impairing CD8^+^ T-cell function and promoting Treg cell and MDSC recruitment. Exosomal transfer of miRNAs induces fibroblast activation, EMT, and immunotherapy resistance, while the downregulation of tumor-suppressive miRNAs further amplifies tumor progression. Although exosome signatures in nasopharyngeal and oral cancers show diagnostic and prognostic potential, clinically validated biomarkers remain scarce. Clinically, combining PD-1/PD-L1 blockade with strategies that neutralize or reprogram CAF subtypes and their exosomes, such as miRNA inhibitors, metabolic modulators, or engineered exosomes, may overcome immune exclusion and enhance immunotherapy durability in HNSCC. The subtype mapping of CAF exosomes, combined with the better understanding of the achieved mechanisms in targeted tumor and immune cells, will be an important future contribution to this field. This also requires the reliable sorting of exosomes in quantities sufficient for reproducible mechanistic studies.

## Figures and Tables

**Table 1:** Key exosomal miRNAs involved in CAF–tumor crosstalk in HNSCC.

miRNA	Source	Primary Targets	Affected Pathways	Functional Consequences
miR-5100	TEXs	QKI	AKT, STAT3	induces CAF differentiation; ↑ α-SMA; ↑ cytokine secretion; ECM remodeling
miR-21	TEXs; CAF	PTEN, PDCD4	SMAD-dependent signaling, Wnt/β-catenin, JAK/STAT3, non-canonical TGF-β/p38 MAPK	promotes EMT, CAF differentiation, immune evasion (↑ PD-L1); enhances tumor growth and stress response; contributes to metastasis and therapy resistance
miR-196a	CAF	CDKN1B, ING5	cell-cycle regulation, apoptosis pathways	confers cisplatin resistance; promotes EMT and stemness; inhibition restores chemosensitivity
miR-215	TEX; CAF (hypoxia-induced)	RB1, p53-associated regulators	DNA damage response, cell survival pathways	enhances tumor survival; promotes chemoresistance; supports CAF-like differentiation via TGF-β/SMAD activation
miR-192/215	TEXs	Caveolin-1	TGF-β/SMAD	induces CAF-like differentiation of normal fibroblasts; reinforces pro-invasive microenvironment
miR-3188	CAF (↓ in CAFs vs. normal fibroblasts)	BCL2	apoptosis regulation	loss in CAF-Exos promotes proliferation and inhibits apoptosis in tumor cells; miR-3188 supplementation suppresses tumor growth

TEXs: tumor-derived exosomes, CAF: cancer-associated fibroblasts, ECM: extracellular matrix, EMT: epithelial to mesenchymal transition.

## Data Availability

No new data were created or analyzed in this study.

## Supplementary Materials

The following supporting information can be downloaded at https://www.mdpi.com/article/10.3390/cells14241978/s1. Figure S1: Prisma-ScR flow diagram. Table S1: Included original research studies.

## Abbreviations

The following abbreviations are used in this manuscript:

Arg-1	Arginase-1
AKT	Protein kinase B
BCL2	B-cell lymphoma 2
CAFs	Cancer-Associated Fibroblasts
CD	Cluster of Differentiation
CDKN1B	Cyclin-dependent kinase inhibitor 1B
CX3CR1	C-X3-C motif chemokine receptor 1
CXCL	C-X-C motif chemokine ligand
EGF	Epidermal Growth Factor
EMT	Epithelial-to-Mesenchymal Transition
FasL	Fas Ligand
GLUT-1	Glucose transporter type 1
HGF	Hepatocyte Growth Factor
HNCAF	Head and neck cancer-associated fibroblasts
ICIs	Immune checkpoint inhibCX3CR1 blasts
IL	Interleukin
ING5	Inhibitor of Growth Family Member 5
LAG-3	Lymphocyte-activation gene 3
MAPK	Mitogen-activated protein kinase
MHC	Major Histocompatibility Complex
PDCD4	Programmed Cell Death 4
PD-1	Programmed Death 1
PD-L1	Programmed Death-Ligand 1
PTEN	Phosphatase and Tensin Homolog
Rab3D	Ras-related protein Rab-3D
RB1	Retinoblastoma 1
SMAD	Suppressor of Mothers against Decapentaplegic
STAT3	Signal Transducer and Activator of Transcription 3
Th17	T helper 17 cells
TSG101	Tumor Susceptibility Gene 101
TIM-3	T-cell immunoglobulin and mucin-domain containing-3
TIGIT	T-cell immunoreceptor with Ig and ITIM domains
UICC	Union for International Cancer Control
WNT	Wingless-type MMTV integration site family
