# Supplementary material for: Cancer-Associated Fibroblasts-Derived Exosomes as Mediators of Immunotherapy Resistance in Head and Neck Squamous Cell Carcinoma
Source: Cells. 2025 Dec 12;14(24):1978. doi: 10.3390/cells14241978 (PMC12732283; doi:10.3390/cells14241978)

Supplementary Figure S1: Prisma-ScR flow diagram illustrating the study selection process for this review.

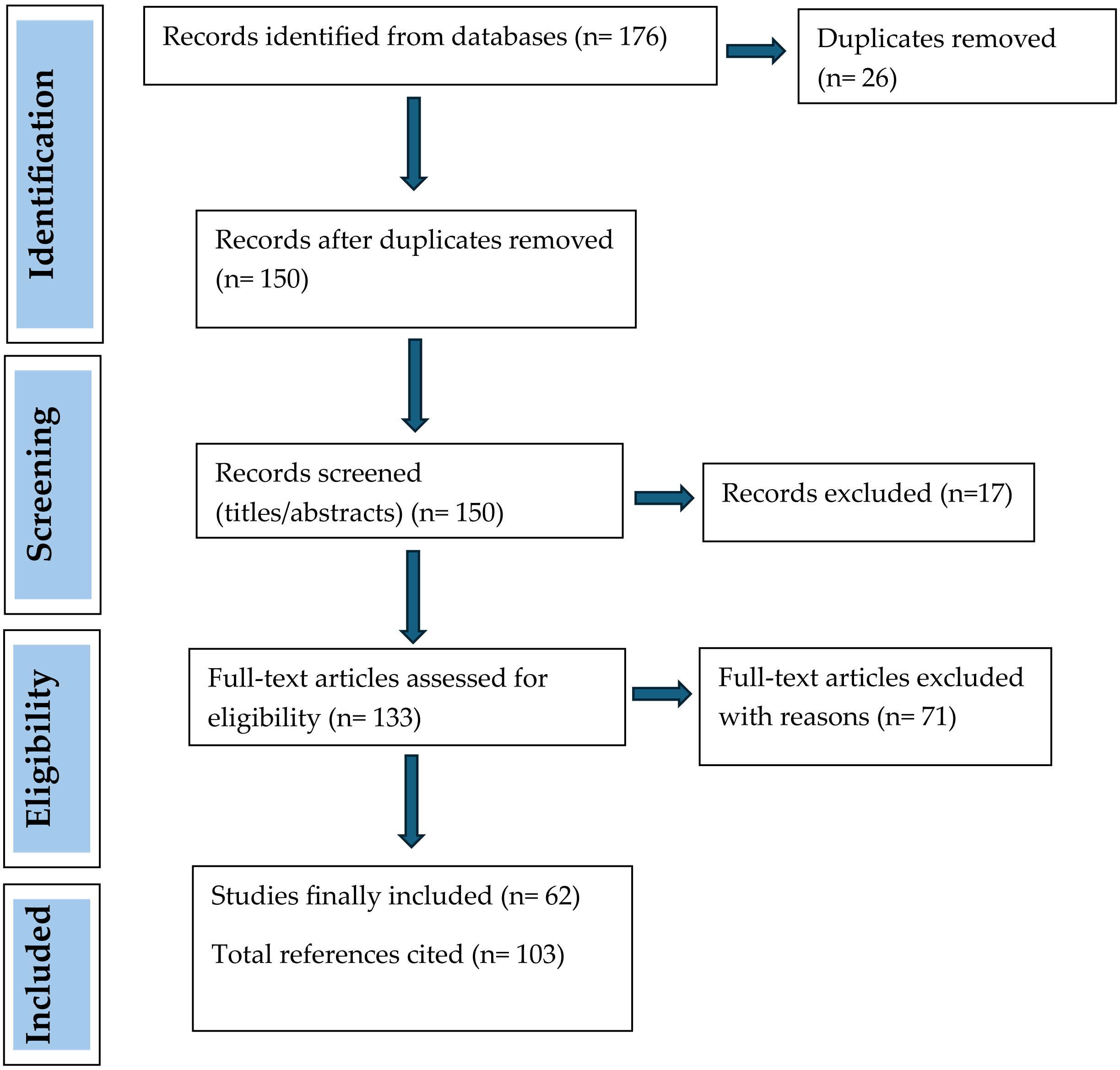

Supplement: Supplementary file 1 [file cells-14-01978-s001.zip › cells-3996676-supplementary/Supplementary Figure S1.pdf]
