# Supplementary material for: Cancer-Associated Fibroblasts-Derived Exosomes as Mediators of Immunotherapy Resistance in Head and Neck Squamous Cell Carcinoma
Source: Cells. 2025 Dec 12;14(24):1978. doi: 10.3390/cells14241978 (PMC12732283; doi:10.3390/cells14241978)
Supplement: Supplementary file 1 [file cells-14-01978-s001.zip › cells-3996676-supplementary/Supplementary Table S1.pdf]

Supplementary Table S1: Overview of all included original research studies (n=62).

No.	Year	Author	Model	Key findings
1	2000	Freeman et al.	in vitro	Identified PD-1 engagement by a novel B7 family ligand and showed PD-1 negatively regulates lymphocyte activation
2	2005	Kim et al.	in vitro/clinical	Tumor-derived vesicles from oral cancer patients induced apoptosis of activated T lymphocytes in cell-based assays; immune-suppressive exosome effect
3	2005	Gastpar et al.	in vitro / in vivo	Hsp70-positive tumor exosomes activate NK cell migration and cytolytic activity in cellular assays
4	2009	Parolini et al.	in vitro	Acidic extracellular pH alters exosome release/uptake in tumor cells; mechanism for TME regulation of exosomes
5	2015	Yang et al.	in vitro / in vivo	Rab3D overexpression promotes invasive behavior; mechanistic molecular evidence for tumor metastasis
6	2016	Ferris et al.	clinical trial	Nivolumab improves overall survival vs. standard therapy in recurrent/metastatic HNSCC; established clinical efficacy of PD-1 blockade
7	2016	Steinbichler et al.	in vitro	Tumor-associated fibroblast-conditioned media induces cisplatin resistance in HNSCC cell lines; evidence for CAF-mediated chemotherapy resistance
8	2017	Garcia-Diaz et al.	in vitro	Interferon receptor pathways controlling PD-L1/PD-L2 expression in tumor cells, linking cytokine signaling to checkpoint ligand regulation
9	2017	Ribas et al.	in vivo/preclinical	Oncolytic viruses increase intratumoral T cell infiltration and enhance anti-PD-1 efficacy in tumor-bearing animal models
10	2017	Puram et al.	in vitro	Single-cell RNA-seq of human tumors revealed cellular heterogeneity and TME composition, identifying immune and stromal cell states
11	2018	Liu et al.	in vivo	TIM-3 blockade reduced regulatory T cells and relieved immunosuppression in HNSCC models
12	2018	Bartoschek et al.	in vitro / in vivo	Spatially and functionally distinct CAF subclasses via single-cell sequencing and functional assays
13	2019	Tang et al.	in vitro	Showed tumor-secreted Hsp90α localized to exosome surfaces mediates tumor–stromal communication through autocrine/paracrine mechanisms in cell-based assays

14	2019	Ferruzzi et al.	in vitro	Compressive remodeling of collagen networks changes fluid transport properties in engineered matrices, mechanistic implications for tumor growth
15	2019	Qin et al.	in vitro	CAF-derived exosomal miR-196a induces cisplatin resistance in HNC cells by targeting CDKN1B and ING5 in cell-based experiments
16	2019	Wang et al.	in vitro/in vivo	CAF-derived exosomes show decreased miR-3188, which promotes HNSCC proliferation, migration, and metastasis by upregulating FOXP4; restoring miR-3188 suppresses tumor growth
17	2019	Zhang et al.	in vitro/in vivo	B7-H3 expression in CAFs promotes their survival and anti-apoptotic behavior; enhances cancer cell invasion and metastasis
18	2020	Razzo et al.	in vivo	Tumor-derived exosomes promote carcinogenesis in a murine oral squamous cell carcinoma model
19	2020	Guo et al.	in vitro	Specific exosomal miRNAs mediate drug resistance mechanisms in cancer cell systems
20	2020	Jung et al.	in vitro / clinical data	EMT gene signature is associated with poor prognosis and TME features; analyses used patient datasets plus cell-based validations
21	2020	Jiang et al.	in vivo / in vitro	Anti-SerpinB9 therapy induces direct tumor cell killing and enhances immune-mediated tumor control; SerpinB9 as a promising immunotherapy target
22	2020	Steinbichler et al.	in vitro / clinical samples	Slug acts as an EMT marker in HNSCC, tested in tumor samples and cellular assays
23	2020	Schroeder et al.	in vitro/preclinical	Circulating exosomes from HNC patients suppress B cell proliferation and activity, suggesting an immunosuppressive role in the TME
24	2020	Fabre et al.	in vivo (animal xenografts)	Preclinical models showed the FAP-targeting ADC has activity in chemotherapy- and pembrolizumab-resistant solid tumor models
25	2020	Kieffer et al.	in vitro (single-cell tumor profiling from human specimens)	Fibroblast clusters are associated with immunotherapy resistance using single-cell transcriptomics of human tumors
26	2020	Wu et al.	in vitro / in vivo	EBV LMP1-packaged EVs activate fibroblasts to promote tumor progression via autophagy and metabolic coupling
27	2020	Theodoraki et al.	in vitro	Plasma-derived exosomes from HNC patients inhibit B-cell proliferation and activity in cell-

				based assays — immune suppression by circulating EVs
28	2020	Beccard et al.,	in vitro	Characterized immunosuppressive effects of different plasma-exosome populations on immune cells
29	2020	Hofmann et al.	in vitro	$CD16^{+}$ plasma exosomes in HNSCC correlate with systemic immune cell changes, indicating their potential as a liquid biomarker for disease monitoring.
30	2021	Zeng et al.	in vitro	Developed a microfluidic, label-free method to separate nanoscale particles (relevant to exosome isolation)
31	2021	Zhu et al.	in vitro / in vivo	EVs carrying miR-192/215 mediate hypoxia-induced CAF development in HNSCC
32	2021	Nelhűbel et al.	in vitro / in vivo	EGFR alterations modulate cetuximab response and c-MET inhibitor sensitivity in experimental HNSCC models
33	2021	Lee et al.	in vivo	Crosstalk between HNC cells and lymphatic endothelial cells via CXCL5–CXCR2 promotes metastasis
34	2022	Ingruber et al.	in vitro	Transcription factor Slug and protein stabilization mechanisms (HSP70 upregulation) are involved in cadherin switching in HNSCC cell models
35	2022	Dou et al.	in vitro	CAF-derived exosomes suppress immune function through miR-92/PD-L1 pathway using breast cancer cell and immune assays
36	2022	Obradovic et al.	in vitro	Identified CAF subpopulations that are immunostimulatory; their presence correlated with better response to immunotherapy; predictive biomarkers for HNSCC immunotherapy response
37	2022	Hofmann et al.	clinical	Saliva-derived exosome cargo in HNC patient; identified potential diagnostic biomarkers from patient samples
38	2022	Hofmann et al.	clinical/in vitro	Plasma-derived exosomes from HNC patients have treatment-dependent effects on EMT in 40cell assays, using patient plasma and cell-based readouts
39	2023	Theodoraki et al.	clinical	Reported CD16+ plasma exosomes correlate with peripheral blood monocyte changes in HNC patients, indicating clinical biomarker associations
40	2023	Tengler et al.	ex vivo / translational research	Plasma-derived small extracellular vesicles from HNC patients promote angiogenesis, enhancing tumor blood vessel formation

41	2023	Federspiel et al.	in vitro	p38 MAPK inhibition targets mesenchymal-transdifferentiated tumor cells in HNSCC
42	2023	Mito et al	in vitro / in vivo	Tumor exosomes activate CAFs and shape inflammatory TME in HNSCC
43	2023	Szabo et al.	in vitro / computational	CAFs are major contributors to EMT signatures in the TME
44	2023	Taddio et al.	in vivo (Preclinical animal study)	Therapeutic potential of radiolabeled FAPI-46 in mouse soft-tissue sarcoma models
45	2023	Theodoraki et al.	in vitro	Plasma-derived small EVs from HNC patients promote angiogenesis in cell-based angiogenesis assays
46	2023	Federspiel et al.	in vitro	Patient-derived CAFs enhance tumor cell colonization after radiotherapy
47	2023	Hoffmann et al.	clinical	CTLA4 promoter methylation associates with CTLA-4 expression and predicts immunotherapy response in HNSCC patient samples
48	2023	Walsh et al.	in vivo	CXCL5 depletion improved T-cell infiltration and response to anti-PD-1 therapy
49	2023	Ye et al.	in vitro / in vivo	Hypoxic HNSCC cells secrete exosomes enriched by miR-21, which activate CAFs via PTEN/AKT pathway suppression. Activated CAFs enhance tumor invasion and metastasis; blocking exosomal miR-21 reduces CAF activation and metastatic spread
50	2024	Theodoraki et al.	in vivo	Plasma-derived exosomes from HNC patients induce type-2 like macrophage polarization and elevated CXCL4 secretion in monocyte-derived macrophages
51	2024	Taddio et al.	in vivo/preclinical	[225Ac]Ac-FAPI-46 in mouse models of soft-tissue sarcoma showing tumor regression and limited off-target toxicity
52	2024	Li et al.	clinical / in vivo	Spatial and single-cell transcriptomics on human HNSCC identified a CAF subset that restricts CD8+ T-cell infiltration and antitumor activity
53	2024	Duan et al.	in vitro / in vivo	Hypoxia-induced miR-5100 in tumor exosomes activates CAFs and promotes metastasis in HNSCC models
54	2024	Jiang et al.	in vivo	Anti-SerpinB9 therapy leads to direct tumor killing and enhances immunotherapy efficacy
55	2024	Hansen et al.	clinical	Phase Ib clinical trial showing safety and preliminary activity of an immunocytokine combined with cetuximab in HNSCC patients
56	2025	Uppaluri et al.	clinical	Large clinical study demonstrating effects of pembrolizumab in neoadjuvant/adjuvant

				settings for locally advanced HNC; reporting clinical outcomes and safety
57	2025	Ren et al.	in vivo / in vitro	ApCAFs increase the CD4/CD8 T-cell ratio to promote tumor growth; inhibiting MIF or STAT3 reduces apCAF formation and tumor progression
58	2025	Peyraud et al.	clinical	Identified two CAF subsets ($FAP^+/\alpha SMA^+$ and $MYH11^+/\alpha SMA^+$) associated with primary resistance to immune checkpoint inhibitors, linked to $CD8^+$ T cell exhaustion and increased regulatory $CD4^+$ T cell infiltration
59	2025	Walsh et al.	in vivo	Depletion of CXCL5 from tumor cells increased T cell infiltration and enhanced the efficacy of anti-PD-1 therapy, indicating CXCL5 as a therapeutic target
60	2025	Wu et al.	in vitro and in vivo	EVs loaded with a GLUT1 inhibitor reduce extracellular matrix stiffness and remodel the TME; this enhances T-cell infiltration
61	2025	Huber et al.	in vitro	Plasma sEVs from HNSCC patients reprogram macrophages into metastasis-promoting TAMs, suppress T cell activation / survival, and induce a macrophage-mediated niche that promotes tumor cell chemotaxis and reverses EMT in tumour cells
62	2025	Groupe Oncologie Radiotherapie Tete et Cou	clinical	Adjuvant nivolumab added to cisplatin-radiotherapy improves disease-free survival in high-risk HNSCC

TME: Tumor Microenvironment; EMT: Epithelial-to-Mesenchymal Transition; HNSCC: Head and Neck Squamous Cell Carcinoma; HNC: Head and Neck Cancer; CAF: Cancer-Associated Fibroblasts
